# Supplementary material for: African American Women with Cardiometabolic Complications of Pregnancy Have Decreased Serum Abundance of Specialized Pro-Resolving Lipid Mediators and Endocannabinoids
Source: Nutrients. 2022 Dec 28;15(1):140. doi: 10.3390/nu15010140 (PMC9823622; doi:10.3390/nu15010140)
Supplement: Supplementary file 1 [file nutrients-15-00140-s001.zip › DHA Paper Figures Dec 2022_Final.pptx]

## Slide 1
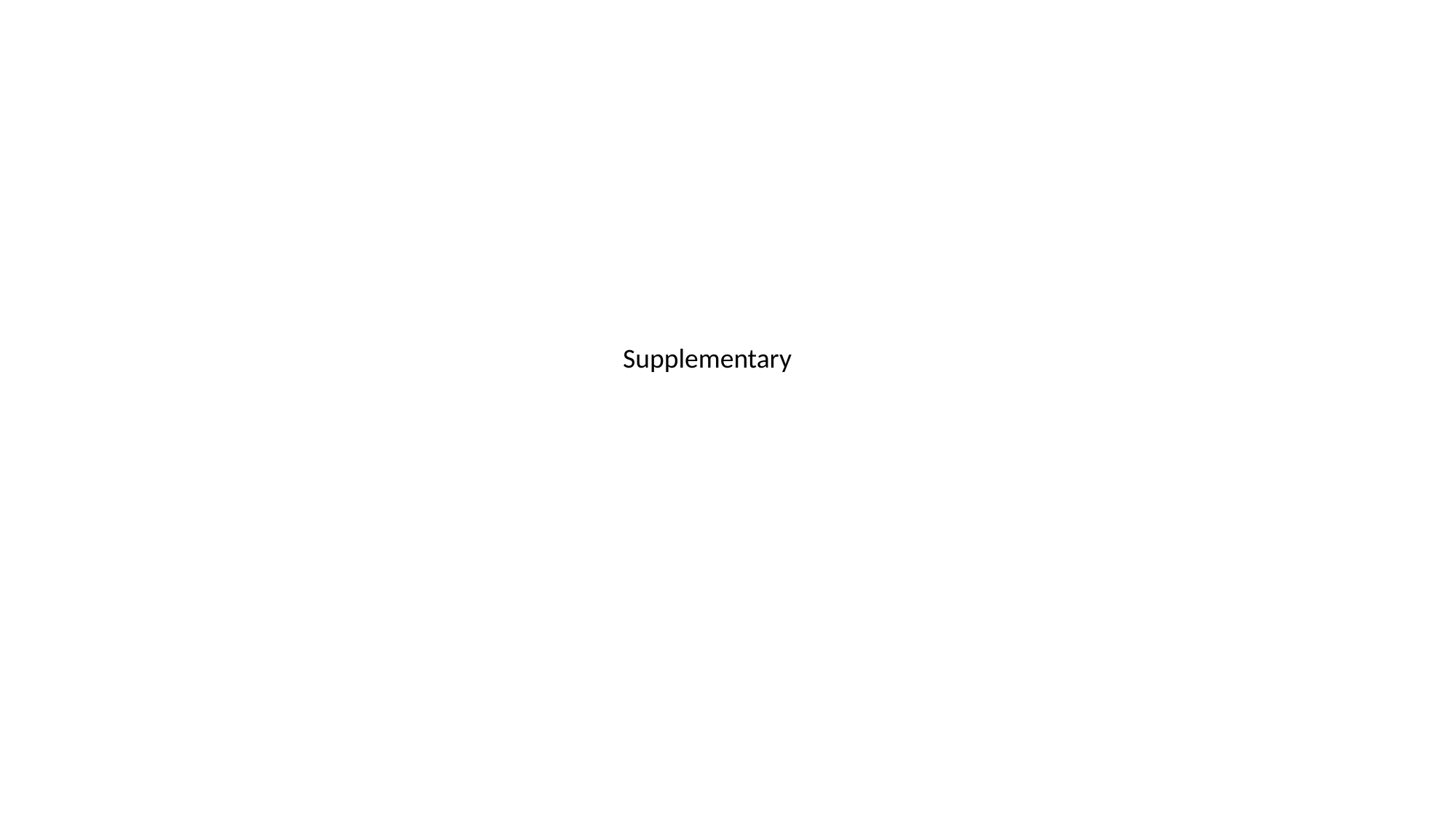

Supplementary

## Slide 2
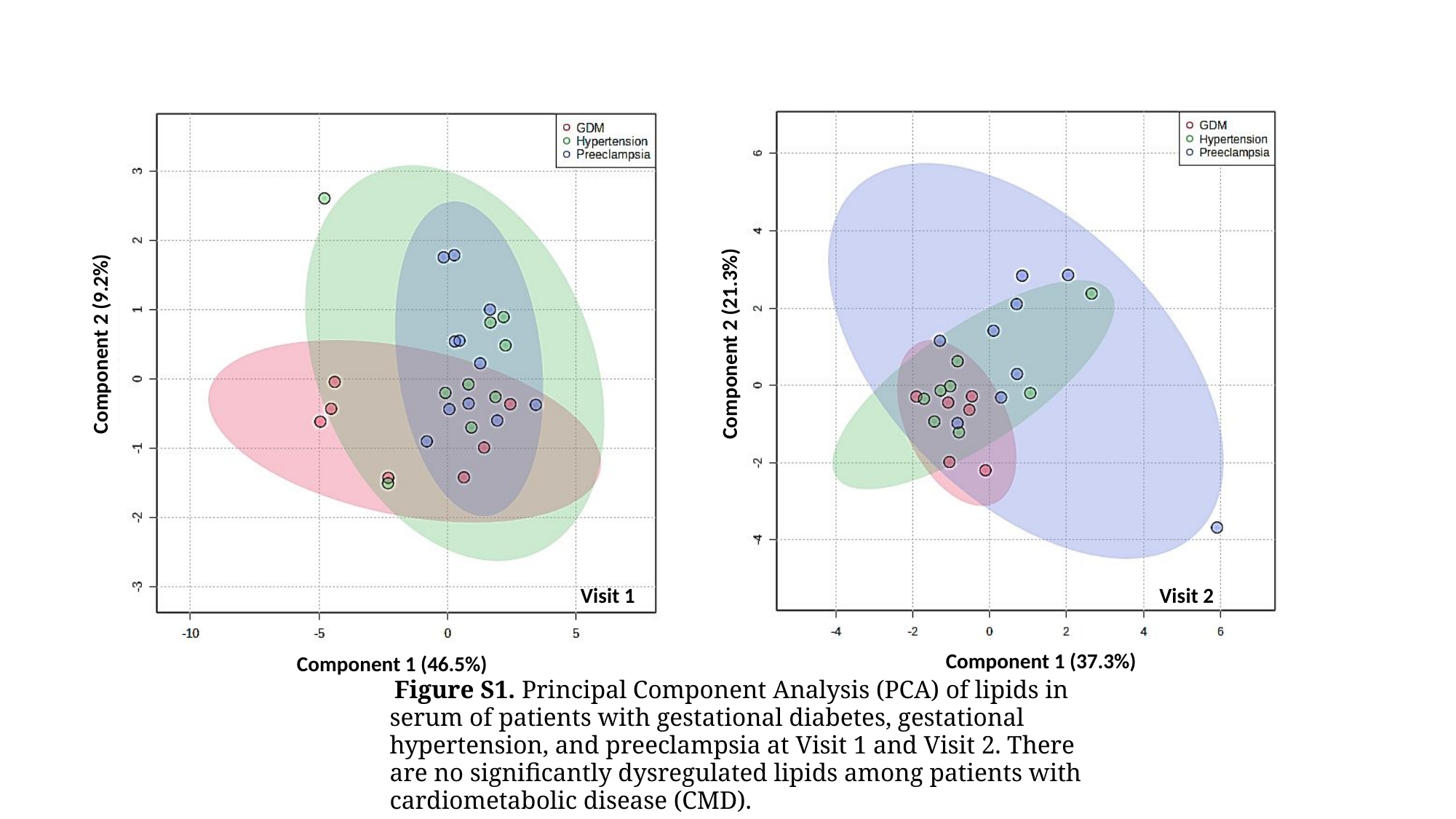

Component 2 (9.2%)
Component 2 (21.3%)
Visit 2
Visit 1
Component 1 (37.3%)
Component 1 (46.5%)
 Figure S1. Principal Component Analysis (PCA) of lipids in serum of patients with gestational diabetes, gestational hypertension, and preeclampsia at Visit 1 and Visit 2. There are no significantly dysregulated lipids among patients with cardiometabolic disease (CMD).

## Slide 3
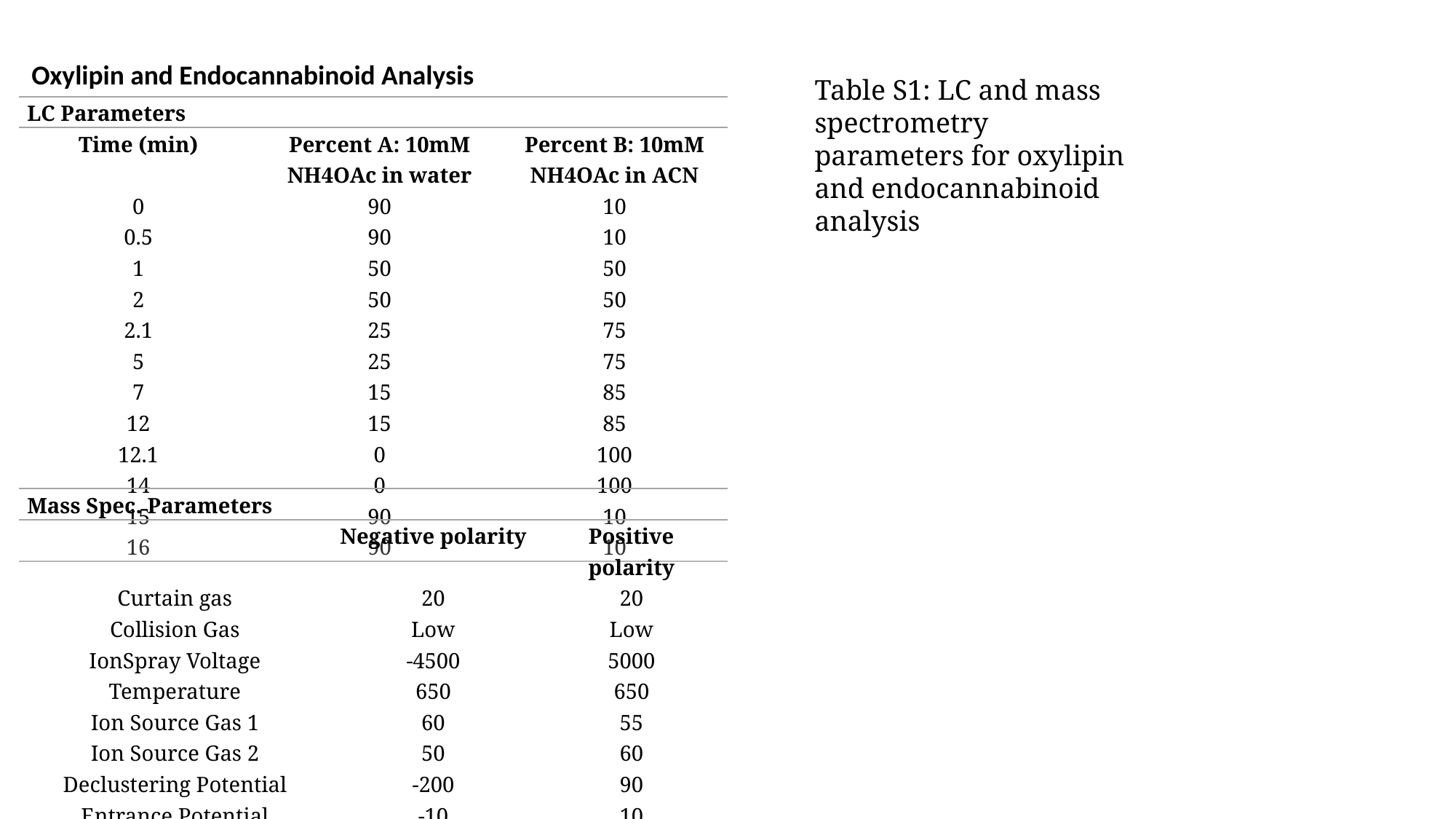

Oxylipin and Endocannabinoid Analysis
Table S1: LC and mass spectrometry parameters for oxylipin and endocannabinoid analysis
| LC Parameters | | |
| --- | --- | --- |
| Time (min) | Percent A: 10mM NH4OAc in water | Percent B: 10mM NH4OAc in ACN |
| 0 | 90 | 10 |
| 0.5 | 90 | 10 |
| 1 | 50 | 50 |
| 2 | 50 | 50 |
| 2.1 | 25 | 75 |
| 5 | 25 | 75 |
| 7 | 15 | 85 |
| 12 | 15 | 85 |
| 12.1 | 0 | 100 |
| 14 | 0 | 100 |
| 15 | 90 | 10 |
| 16 | 90 | 10 |
| Mass Spec. Parameters | | |
| --- | --- | --- |
| | Negative polarity | Positive polarity |
| Curtain gas | 20 | 20 |
| Collision Gas | Low | Low |
| IonSpray Voltage | -4500 | 5000 |
| Temperature | 650 | 650 |
| Ion Source Gas 1 | 60 | 55 |
| Ion Source Gas 2 | 50 | 60 |
| Declustering Potential | -200 | 90 |
| Entrance Potential | -10 | 10 |
| Collision Energy | -40 | 47 |
| Collision Cell Exit Potential | -11 | 18 |

## Slide 4
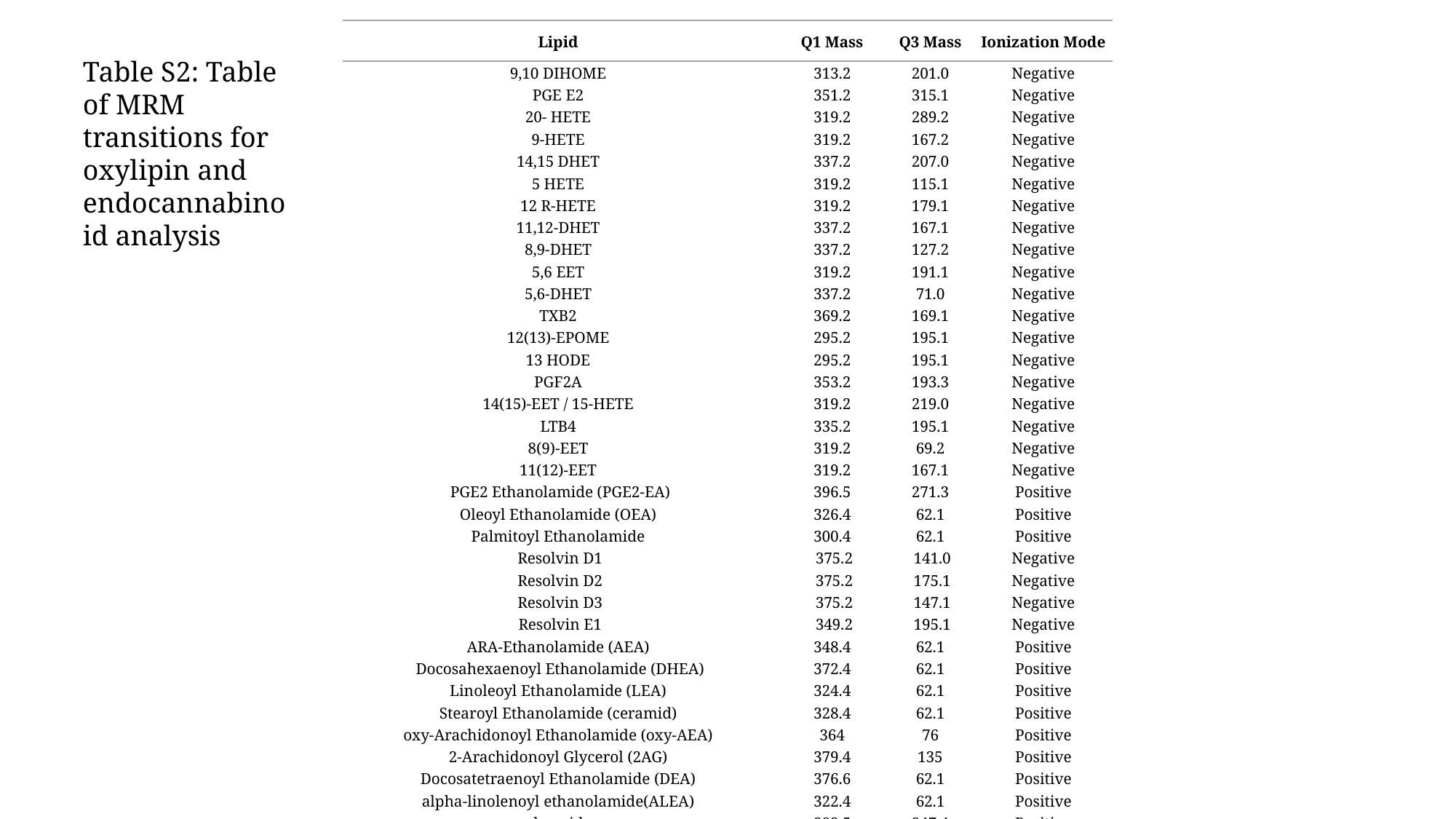

| Lipid | Q1 Mass | Q3 Mass | Ionization Mode |
| --- | --- | --- | --- |
| 9,10 DIHOME | 313.2 | 201.0 | Negative |
| PGE E2 | 351.2 | 315.1 | Negative |
| 20- HETE | 319.2 | 289.2 | Negative |
| 9-HETE | 319.2 | 167.2 | Negative |
| 14,15 DHET | 337.2 | 207.0 | Negative |
| 5 HETE | 319.2 | 115.1 | Negative |
| 12 R-HETE | 319.2 | 179.1 | Negative |
| 11,12-DHET | 337.2 | 167.1 | Negative |
| 8,9-DHET | 337.2 | 127.2 | Negative |
| 5,6 EET | 319.2 | 191.1 | Negative |
| 5,6-DHET | 337.2 | 71.0 | Negative |
| TXB2 | 369.2 | 169.1 | Negative |
| 12(13)-EPOME | 295.2 | 195.1 | Negative |
| 13 HODE | 295.2 | 195.1 | Negative |
| PGF2A | 353.2 | 193.3 | Negative |
| 14(15)-EET / 15-HETE | 319.2 | 219.0 | Negative |
| LTB4 | 335.2 | 195.1 | Negative |
| 8(9)-EET | 319.2 | 69.2 | Negative |
| 11(12)-EET | 319.2 | 167.1 | Negative |
| PGE2 Ethanolamide (PGE2-EA) | 396.5 | 271.3 | Positive |
| Oleoyl Ethanolamide (OEA) | 326.4 | 62.1 | Positive |
| Palmitoyl Ethanolamide | 300.4 | 62.1 | Positive |
| Resolvin D1 | 375.2 | 141.0 | Negative |
| Resolvin D2 | 375.2 | 175.1 | Negative |
| Resolvin D3 | 375.2 | 147.1 | Negative |
| Resolvin E1 | 349.2 | 195.1 | Negative |
| ARA-Ethanolamide (AEA) | 348.4 | 62.1 | Positive |
| Docosahexaenoyl Ethanolamide (DHEA) | 372.4 | 62.1 | Positive |
| Linoleoyl Ethanolamide (LEA) | 324.4 | 62.1 | Positive |
| Stearoyl Ethanolamide (ceramid) | 328.4 | 62.1 | Positive |
| oxy-Arachidonoyl Ethanolamide (oxy-AEA) | 364 | 76 | Positive |
| 2-Arachidonoyl Glycerol (2AG) | 379.4 | 135 | Positive |
| Docosatetraenoyl Ethanolamide (DEA) | 376.6 | 62.1 | Positive |
| alpha-linolenoyl ethanolamide(ALEA) | 322.4 | 62.1 | Positive |
| oleamide | 282.5 | 247.4 | Positive |
| dihomo-gamma-linolenoyl ethanolamide | 350.4 | 62.1 | Positive |
| PGE-2 Glycerol Ester | 427.6 | 297.1 | Positive |
Table S2: Table of MRM transitions for oxylipin and endocannabinoid analysis

## Slide 5
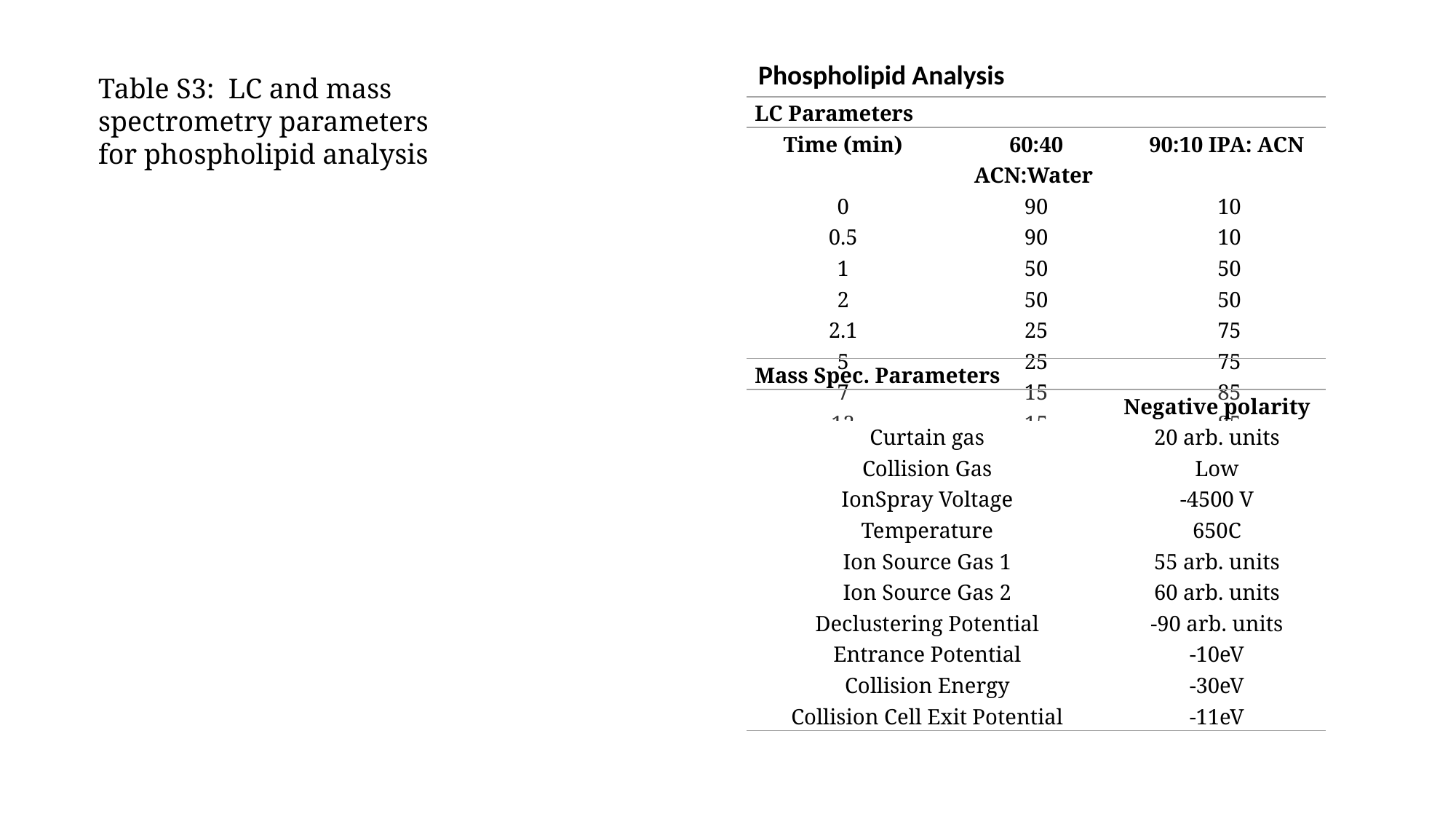

Phospholipid Analysis
Table S3: LC and mass spectrometry parameters for phospholipid analysis
| LC Parameters | | |
| --- | --- | --- |
| Time (min) | 60:40 ACN:Water | 90:10 IPA: ACN |
| 0 | 90 | 10 |
| 0.5 | 90 | 10 |
| 1 | 50 | 50 |
| 2 | 50 | 50 |
| 2.1 | 25 | 75 |
| 5 | 25 | 75 |
| 7 | 15 | 85 |
| 12 | 15 | 85 |
| Mass Spec. Parameters | |
| --- | --- |
| | Negative polarity |
| Curtain gas | 20 arb. units |
| Collision Gas | Low |
| IonSpray Voltage | -4500 V |
| Temperature | 650C |
| Ion Source Gas 1 | 55 arb. units |
| Ion Source Gas 2 | 60 arb. units |
| Declustering Potential | -90 arb. units |
| Entrance Potential | -10eV |
| Collision Energy | -30eV |
| Collision Cell Exit Potential | -11eV |
